# Stakeholder outcome prioritization in the Biologic Abatement and Capturing Kids' Outcomes and Flare Frequency in Juvenile Spondyloarthritis (BACK‐OFF JSpA) trial

**DOI:** 10.1111/hex.13655

**Published:** 2022-11-17

**Authors:** Emily Neu, Cora Sears, Timothy Brandon, Melanie Kohlheim, Jenny Leal, Kweli Archie, English Holland, Miles Holland, Aamena Hameed, Asad Khan, Lynn Murphy, Sean Murphy, Antoinette Neu, Jerome Neu, Justin Neu, Rachel Richmond, Dylan Suplee, Theresa Suplee, Christopher B. Forrest, Pamela F. Weiss

**Affiliations:** ^1^ Department of Pediatrics, Division of Rheumatology, Clinical Futures: A CHOP Research Institute Center of Emphasis Children's Hospital of Philadelphia Philadelphia Pennsylvania USA; ^2^ BACK‐OFF JSpA Research Partners Group Children's Hospital of Philadelphia Philadelphia Pennsylvania USA; ^3^ Department of Pediatrics and Epidemiology, Perelman School of Medicine University of Pennsylvania Philadelphia Pennsylvania USA

**Keywords:** patient‐reported outcomes, spondyloarthritis, stakeholder, trial, tumour necrosis factor inhibitors

## Abstract

**Background:**

The Biologic Abatement and Capturing Kids’ Outcomes and Flare Frequency in Juvenile Spondyloarthritis (BACK‐OFF JSpA) study is a randomized, pragmatic trial investigating different tumour necrosis factor inhibitor de‐escalation strategies for children with sustained inactive disease. In this project, we elicited concept rankings that aided in the selection of the patient‐reported outcome (PRO) measures that should be examined as part of the BACK‐OFF JSpA trial.

**Methods:**

We conducted a discrete choice experiment to evaluate individuals' preferences regarding PROs. Stakeholders assessed a discrete list of 21 outcome concepts, each of which had a Patient‐Reported Outcome Measurement Information System (PROMIS) measure associated with it. PROMIS measures are self‐ or proxy‐reported instruments that are universally applicable to the general population and all chronic conditions. Stakeholders were required to make choices instead of expressing the strength of a preference.

**Results:**

Fourteen caregivers, 12 patients (9–22 years old), 16 rheumatologists and three executives from health insurance companies completed the exercise, which took approximately 10 min. The discrete choice experiment resulted in an estimate of the relative importance of each outcome and rank. All stakeholder groups agreed that the primary PRO should be ‘Pain Interference’, a measure that evaluates the effect of pain on a child's everyday activities, including its impact on social, emotional, mental and physical functioning. Patients and caregivers were mostly aligned in their top priorities, with patients valuing physical health (50% of the top 10) whereas caregivers were more interested in mental health (60% of the top 10). Rheumatologists and health insurance executives were most interested in physical health outcomes, which were ranked 80% and 60% of their top 10 PROs, respectively. Overall, the patients had the most diverse set of prioritized outcomes, including at least one of each category in their top 10 rank order of importance. Patients were also the only stakeholders to prioritize ‘social’ health.

**Conclusions:**

Patients and caregivers were mostly aligned in their outcome priority rankings. The rank‐order list directly informed the creation of a profile of PRO measures for our upcoming trial.

**Patient or Public Contribution:**

Stakeholder partners helped with acquisition of data and lead parent partners helped interpret data.

## 1 BACKGROUND

In 2018, an international task force of pediatric rheumatologists developed recommendations for treating juvenile arthritis to target.^1^ The primary treatment target was the inactive disease, defined as the absence of all clinical signs and patient‐experienced symptoms of inflammatory disease activity. Additionally, the international task force specified several overarching principles for the management of juvenile arthritis which included not only controlling signs and symptoms of disease but also avoidance of drug toxicities and optimization of personal well‐being. Since the introduction of biologic disease‐modifying agents such as tumour necrosis factor inhibitors (TNFi), the inactive disease is a feasible target for children with spondyloarthritis, which accounts for up to 30% of juvenile arthritis. In fact, current treatment approaches for children with spondyloarthritis have resulted in up to 60% attaining inactive disease while on therapy.^2^, ^3^, ^4^

However, there is no information to inform decisions regarding tapering (increasing the time between doses) or stopping TNFi after the inactive disease is achieved. The Biologic Abatement and Capturing Kids' Outcomes and Flare Frequency in Juvenile Spondyloarthritis (BACK‐OFF JSpA) Trial is a randomized pragmatic trial that will improve the evidence base that patients, caregivers and rheumatologists use to make shared decisions about continued treatment versus de‐escalation of therapy in children with spondyloarthritis who have the inactive disease. Unless there is high‐quality and unbiased evidence on TNFi de‐escalation experiences, pediatric patients with spondyloarthritis and their caregivers will not be able to make decisions that take into account the outcomes that are most important to them. In fact, the Outcome Measures in Rheumatology (OMERACT) updated core domain set for studies in juvenile idiopathic arthritis includes patient‐reported outcomes (PROs) like pain, physical function and patient perception of disease activity.^5^ Timing and risk of flare is critical for patients and caregivers to know when making informed decisions about potential de‐escalation of TNFi therapy. The BACK‐OFF JSpA trial will not only determine the timing and risk of disease flare but also the lived experiences of patients undergoing the various treatment strategies. The growing importance of the patients’ lived experiences in clinical research such as the BACK‐OFF JSpA trial is underscored by the US government's Patient Protection and Affordable Care Act that created the Patient‐Centered Outcomes Research Institute,^6^ the creation of the NIH Patient‐Reported Outcomes Measurement Information System® (PROMIS®) initiative,^7^ and the Food and Drug Administration's mandate to use these assessments for medical product labelling claims.^8^

Prior mixed‐methods work from Horton and colleagues has shown that when making decisions about stopping medication, caregivers and patients with juvenile arthritis consider the risk from both the disease and treatment.^9^ Participants emphasize the importance of how their underlying arthritis and treatments aided or hindered with a sense of ‘normalcy’ and safety and also the uncertainty regarding risk of future treatment effects/harms and risk of flare. Ultimately, participants' decisions were informed by trust in their physician and alternate sources of information including social media. In another study consisting of web‐based surveys and focus groups, research themes prioritized by patients and caregivers of children with rheumatic disease included disease flare and medication side effects.^10^ A study by Moser and colleagues underscored the importance of considering relevant PRO measures over the course of juvenile arthritis.^11^ Additionally, several key concepts were elucidated: in this study (1) youth did not feel adequately informed about the purpose of collecting or value of patient‐reported outcomes, (2) assessments used during routine care—in particular those related to function—were outdated and not pertinent to current issues and (3) youth should be involved in the development/selection of instruments to ensure relevance.

The BACK‐OFF JSpA trial team includes a Research Partners Group consisting of members from across the United States who are JSpA patients, caregivers, foundation representatives, payor partners and an adult and pediatric rheumatologist. The Research Partners Group participated in a family studio to help the investigator team understand the trial design preferences of patients and parents within the juvenile SpA population. The family studio provided Research Partners Group members with an opportunity to offer suggestions and voice concerns based on their own experiences. The exercise reported herein aimed to capture the PROs most important to the patient and caregiver stakeholders for inclusion as the primary and secondary outcomes of the second aim of the BACK‐OFF JSpA trial.

## 2 METHODS

### 2.1 Subjects

This was a prospective cross‐sectional study of juvenile SpA stakeholders. Subjects were a convenience sample including the BACK‐OFF JSpA Research Partner Group members (patients, caregivers, foundation and payor representatives), patients and caregivers of children with SpA being treated at the Children's Hospital of Philadelphia, and site investigators for the BACK‐OFF JSpA trial. Eligibility requirements for patient and caregiver stakeholders included either membership on the BACK‐OFF JSpA Research Partners Group or a patient or caregiver of a patient fulfilling all of the following: (1) Diagnosis of juvenile SpA, (2) age 7 years or above, (3) current treatment with a TNFi and (4) evaluated in a rheumatology clinic at the Children's Hospital of Philadelphia in January 2021. Rheumatologists were either members of the BACK‐OFF JSpA Research Partners Group or a Site Investigator for the BACK‐OFF JSpA trial who treats patients with JSpA. National organization (foundation) stakeholders were active organization members that advocate and support individuals living with JSpA. Similarly, payor partner stakeholders were insurance company representatives that provide medical coverage for patients with JSpA. There was no compensation for participation in the survey. The Children's Hospital of Philadelphia Committee for the Protection of Human Subjects approved the protocol for the conduct of this study (IRB 20‐018224) and consent or assent, as appropriate, was obtained from all participants.

### 2.2 Patient‐reported outcomes

We aimed to have participants choose the primary and secondary PROs of most importance from a list of 21 health and wellbeing concepts, each of which had an associated PROMIS tool (https://www.healthmeasures.net/explore-measurement-systems/promis/intro-to-promis/list-of-pediatric-measures). PROMIS measures are self‐ or proxy‐reported instruments designed for use in the general population and across all chronic conditions.^12^ Each PROMIS domain is composed of a collection of items called an ‘item bank’ which encompasses the full range of the latent variable being evaluated. A ‘short form’ is a selection of items that represent the item bank with fewer questions, typically four to eight items. Short forms can be easily administered on mobile devices and are easy to complete. Youths 8–17 years of age can complete self‐report instruments and caregivers can complete parent proxy‐report instruments. The PROMIS short‐forms have been validated in children with juvenile arthritis.^13^

### 2.3 Discrete choice experiment

The discrete choice experiment was pilot tested with local pediatric rheumatologists and research assistants and several stakeholder parent partners. The survey was administered electronically (iPad, smartphone, or computer) using Sawtooth Software either in the office at the time of rheumatology assessment or remotely through emailed invitation.^8^ The experiment was a user‐friendly, quantitative, choice‐based approach to evaluate individuals’ preferences regarding the importance of potential PROs. Sawtooth Software uses empirical Bayes to conduct relative comparisons among the items in the study and provide individual‐level score estimates.^14^ With this software, stakeholders assessed 21 outcomes. If both a caregiver and patient were completing the survey, they were instructed to complete it independently. In preparation for the exercise caregivers and patients were provided with a list of the outcomes under consideration and an explanation of what each measure.

Outcome concepts were drawn from physical, mental, social and global health dimensions (Table 1).

**Table 1:** Pediatric outcomes by category

Dimension	Outcome
Global	Global health
Mental	Life satisfaction Cognitive function Sense of life's meaning Depressive symptoms Stress Anxiety symptoms Positive mood Angry mood or irritability
Physical	Pain interference Mobility Pain behaviours Physical activity Upper extremity function Fatigue Impact on strength activities Sleep‐disturbance Sleep‐related daytime impairment Physical responses to stress
Social	Family relationships Peer‐relationships

*Note*: All domains are measurable by PROMIS pediatric instruments.

**Figure 1:**
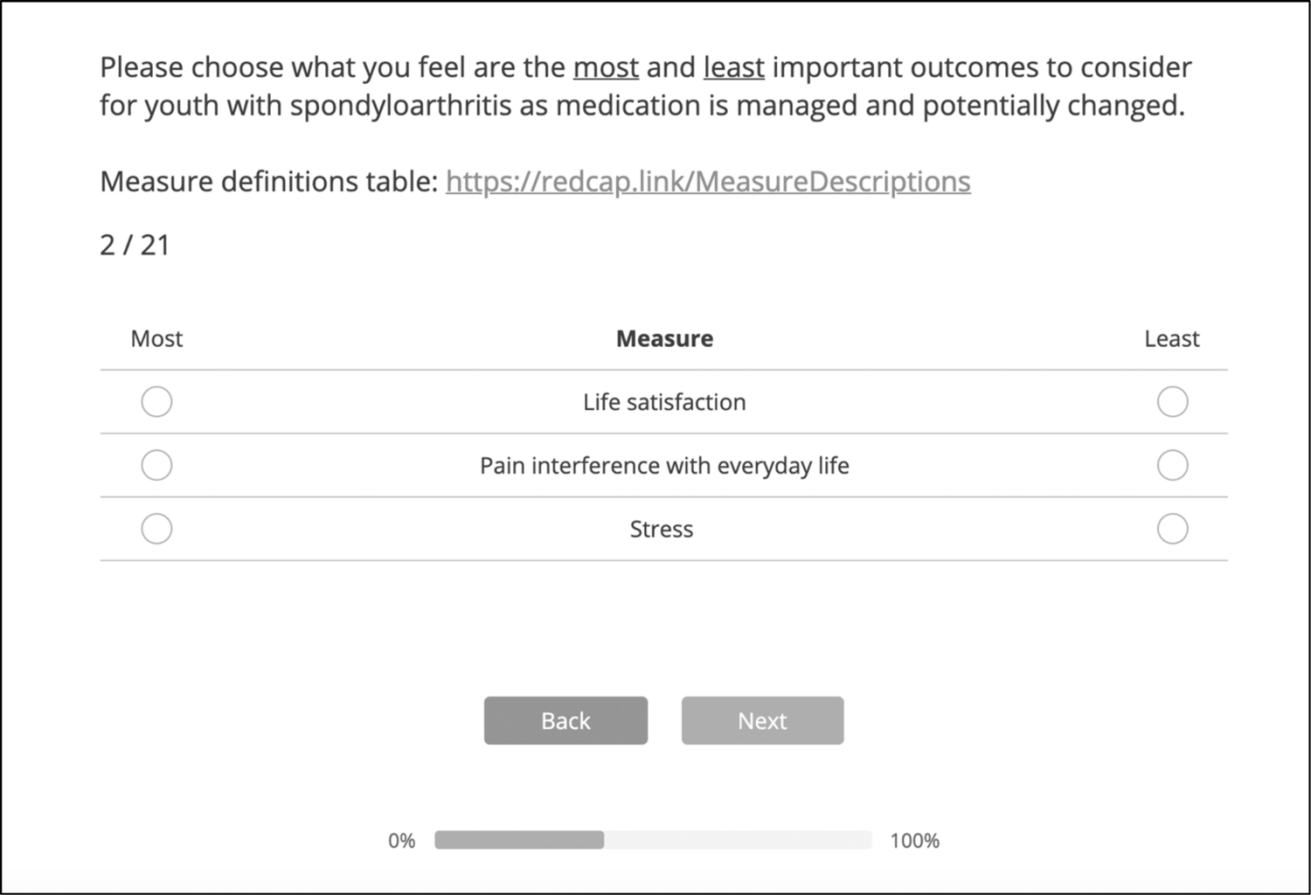
Discrete choice experiment sample question (reproduced with permission from Sawtooth Software, Inc.)

Rather than asking stakeholders to rate all items at once, three outcome concepts were presented at a time (Figure 1). Within each set, stakeholders were instructed to ‘Please choose what you feel are the most and least important outcomes to consider for youth with spondyloarthritis as medication is managed and potentially changed’. This process was repeated for 21 unique outcome combinations, with each outcome being shown three times. Stakeholders were required to make choices instead of expressing the strength of a preference (as would be done with a Likert scale or Delphi rating process). Using this process left no opportunity for scale use bias, where respondents often rate different attributes similarly.

## 3 RESULTS

Fourteen caregivers, 11 patients (ages 9–22 years old), 16 rheumatologists and three executives from health insurance companies completed the exercise in a median time of 10.2 min (interquartile range: 7.3–14.0 min). For patient stakeholders (*n* = 11), the average age was 16 years, 91% were White and seven identified as male and four as female. Half of the rheumatologists identified as female, while all of the payor representatives (*N* = 3) identified as male. Caregivers were predominately female (13 females, one male) and were the most racially diverse group of stakeholders with 21% of respondents reporting their race as non‐White.

**Figure 2:**
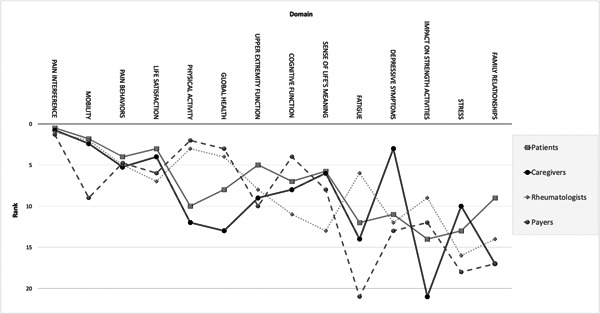
Stakeholder rank order of importance by domain

The discrete choice experiment resulted in an estimate of the relative importance of each outcome and rank (Table 2, Figure 2). All stakeholder groups agreed that the PROMIS Pain Interference measure was the most important to consider during TNFi therapy de‐escalation. This measure evaluates the effect of pain on a child or adolescent's everyday activities, including its impact on social, psychological and physical functioning. All groups except for payors ranked Mobility as the second most important outcome. Payors ranked Mobility ninth.

**Table 2:** Stakeholder estimates of relative importance

Pediatric domains	Relative importance				
	All	Patients	Caregivers	Rheumatologists	Payors
Pain interference	10.9	9.8	11.2	11.4	10.9
Mobility	9.2	8.9	8.4	10.6	5.8
Pain behaviours	7.2	7.1	6.4	8.0	7.1
Life satisfaction	6.9	8.3	6.5	6.1	7.1
Physical activity	6.5	4.8	4.3	8.7	10.5
Global health	6.5	5.8	4.1	8.6	7.7
Upper extremity function	5.6	6.1	4.9	6.0	5.3
Cognitive function	4.8	5.8	5.2	3.4	7.4
Sense of life's meaning and purpose	4.8	5.9	6.4	2.5	5.9
Fatigue	4.8	3.9	4.1	6.7	0.3
Depressive symptoms	4.6	4.1	6.7	3.3	3.8
Impact on strength activities	3.4	3.2	1.8	4.7	3.9
Stress	3.4	3.4	4.9	2.5	1.6
Family relationships	3.2	5.5	2.6	2.5	1.8
Anxiety symptoms	3.0	3.2	5.4	1.4	1.1
Positive mood	3.0	3.0	4.3	2.0	2.0
Sleep‐disturbance	2.7	1.5	2.5	3.6	4.0
Peer‐relationships	2.6	2.9	3.0	1.8	3.4
Sleep‐related daytime impairment	2.5	1.9	2.1	2.5	6.5
Physical responses to stress	2.5	2.5	3.0	1.8	3.2
Angry mood or irritability	2.0	2.4	2.2	1.8	0.6

**Figure 3:**
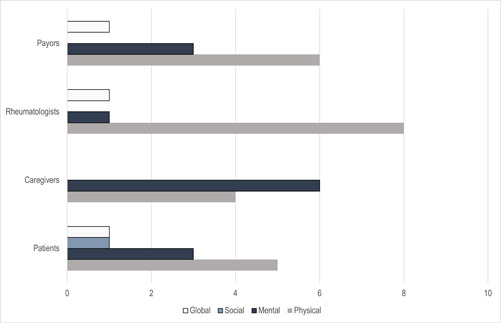
Health category of top 10 domains selected by stakeholder type

After Pain Interference and Mobility, patients’ next priority was Life Satisfaction. The top 10 pediatric domain categories by stakeholder type are shown in Figure 3. Overall, patients highly valued physical health, with 50% of their top 10 categorized as physical health outcomes. Of all stakeholder groups, the patients selected the most diverse set of outcomes, which included at least one outcome from global, mental, social and physical health in their top 10 rank order of importance. Patients were the only stakeholder group to prioritize an outcome (family relationships) from the social health category.

Caregivers highly valued mental health, with six mental health outcomes occupying the top 10. After Pain Interference and Mobility, the next outcomes prioritized by this group were Depressive Symptoms, Life satisfaction and a Sense of Life's Meaning. Rheumatologists and payors valued physical health the most, which included 80% and 60% of their top 10, respectively. Following Pain Behaviours and Mobility, rheumatologists ranked Physical Activity and Global Health as the most important.

## 4 DISCUSSION

In this study, we asked patients, caregivers, rheumatologists and payor partners to rate the importance of specific patient‐reported outcomes that were being considered for an upcoming trial evaluating three therapy de‐escalation strategies for children with SpA who have achieved sustained inactive disease for at least 6 months. We found that the top priority for patients, caregivers and rheumatologists was Pain Interference, which measures the impact of pain on multiple dimensions of functioning and as per PROMIS classification, it was assigned to physical health. Of the stakeholder groups, patients were the only ones to include at least one outcome from each of the four dimensions in their top 10 rank order of importance. This underscores the diversity of outcome preferences among patients. Conversely, the rheumatologists were fairly narrow in their view, rating multiple physical health outcomes highly. These results emphasize the importance of including multiple perspectives in outcome prioritization and ultimately assessment, which aligns with the updated JIA Core Domain Set following the OMERACT methodology.^5^ Our results also underscore findings from a recent qualitative study of youth with juvenile arthritis that concluded that youth must be involved in outcome choice to ensure relevance.^11^

This study has both strengths and limitations. The strengths include stakeholders from pediatric academic centres from across the United States, a relatively efficient data collection process that allowed respondents to complete the survey in about 10 min, and very few missing data points. A limitation of the study is the relatively small sample size, especially when stratified by stakeholders which limits the generalizability of these findings. It is possible that patients and parents who are not members of the BACK‐OFF JSpA Research Partners Group or survey nonrespondents have different values and perspectives on what PROs are most important to consider in a de‐escalation trial. However, in the updated JIA Core Domain Set^5^ which included a large international sample of stakeholders, pain, physical health and overall well‐being was voted as mandatory outcomes in all trials and consistent with our findings. Finally, most but not all BACK‐OFF JSpA site investigators or Research Partner Group members completed the survey. It is unknown what differences, if any, exist between survey respondents versus nonrespondents.

With these strengths and limitations in mind, our findings underscore the importance of stakeholder involvement in study design. Patient and caregiver stakeholders are an integral part of the investigative team for the BACK‐OFF JSpA trial. If patients and caregiver stakeholders had not done this exercise, the PRO measures that would have been investigated, based upon physician prioritization, would not have been in direct alignment with what the patients and caregivers are truly most interested in. Specifically, if the PRO profile was developed with input only from clinicians the profile would still have included pain interference, mobility and pain behaviours—albeit in a slightly different priority order‐ however global health would have been included rather than life satisfaction. Since PROs are now part of the updated OMERACT JIA core set,^5^ a strong argument could be made that all trials in JIA should incorporate input from patients, caregivers and clinicians into the design and/or conduct of the study. Depending upon the question being studied in each trial or study, the PROs prioritized by alternative stakeholder groups are likely to differ. As it relates to the BACK‐OFF JSpA trial if the risk of disease flare is only marginally different between the treatment strategies being studied, differences in PROs could be tremendously informative for shared decision‐making regarding which strategy patients and caregivers will ultimately prefer.

Our findings highlight the importance of collecting patient and caregiver preferences on study questions during the planning stages of a trial. The rank‐order list from the patient and parent caregiver stakeholders from this exercise directly informed the primary and secondary PROs for the upcoming BACK‐OFF JSpA trial with the primary patient‐reported outcome being Pain Interference and the secondary outcomes being Mobility, Life Satisfaction and Pain Behaviours. Further, we need to learn how these outcomes ultimately influence stakeholders’ interpretation of the results of the upcoming trial and their subsequent point‐of‐care therapy de‐escalation preferences. Our study was designed specifically for the JSpA population being treated with a TNFi who would be potentially eligible for trial enrolment to evaluate therapy de‐escalation. Therefore, our results may not generalize to all juvenile arthritis trials. However, the ease with which this exercise was conducted and our results underscore that similar exercise(s) can, and should be completed for trials of patients with juvenile arthritis at the design phase so that measures and outcomes that are both relevant and highly valued to the patient population under study are included.

## AUTHOR CONTRIBUTIONS

Pamela F. Weiss, Cora Sears and Timothy Brandon helped with the design of work, analysis, interpretation of data and drafting of the manuscript. All authors helped with the acquisition of data. Emily Neu, Melanie Kohlheim and Jenny Leal helped with interpretation of the data. Christopher B. Forrest helped with the design of work. All authors approved the submitted version of the manuscript.

## CONFLICT OF INTEREST

The authors declare no conflict of interest.

## ETHICS STATEMENT

This study's protocol was reviewed and approved by the Children's Hospital of Philadelphia's (IRB 21‐018442) Committees for the Protection of Human Subjects.

## Data Availability

The data that support the findings of this study are available from the corresponding author upon reasonable request.
